# Arsenic in Portuguese Rice: Is There Any Risk?

**DOI:** 10.3390/foods11030277

**Published:** 2022-01-20

**Authors:** Alexandra Silva, André Pereira, Liliana Silva, Angelina Pena

**Affiliations:** LAQV, REQUIMTE, Laboratory of Bromatology and Pharmacognosy, Faculty of Pharmacy, University of Coimbra, Polo III, Azinhaga de Stª Comba, 3000-548 Coimbra, Portugal; alexandra.silv@hotmail.com (A.S.); ljgsilva@hotmail.com (L.S.); apena@ci.uc.pt (A.P.)

**Keywords:** arsenic, rice, environmental contaminants, estimated daily intake, risk assessment

## Abstract

Arsenic is a metalloid with natural and anthropogenic sources and its inorganic form is toxic to humans. Rice is highly consumed worldwide and is prone to arsenic contamination; therefore, this study evaluated the inorganic arsenic content of 70 Portuguese rice samples. These were analysed through inductively coupled plasma mass spectrometry (ICP-MS) with a detection limit of 3.3 µg kg^−1^. The average contamination was of 29.3 µg kg^−1^, with brown and short rice presenting higher values than white and long rice. The highest concentration, 100 µg kg^−1^, equalled the maximum residue limit (MRL) for rice destined for infants’ consumption. The estimated daily intake (EDI) surpassed the benchmark dose (lower confidence limit 10%) (BMDL_10_) of 0.3 µg kg^−1^ of bw/day considering children in the 95th percentile of rice consumption and the worst-case scenario concentration. However, other sources also contribute to the EDI and some population groups can exceed the BMDL_10_.

## 1. Introduction

Modern agriculture practices have increased productivity, but at high environmental costs. The increased use of chemical compounds has led to serious pollution problems across the planet, causing different environmental issues, such as the contamination of metals and metalloids in food chains, jeopardizing food safety. Food and water are the most common sources of human exposure to metals and metalloids [1,2].

Among these toxic compounds is arsenic, a metalloid or semi-metal with several natural sources like minerals, rocks, soils and sediments formed from these arsenic-containing rocks, as well as geothermal and volcanic phenomena. Moreover, it is a chemical element used as a glass clarifier, in fireworks and in the pesticide industry. Arsenic has different chemical forms, including organic and inorganic species, with the later presenting higher toxicity [3,4,5,6].

Inorganic arsenic is considered a carcinogen, belonging to Group 1 of the International Agency for Research on Cancer (IARC), because long-term exposure is associated with an increased risk of several carcinomas, including skin, bladder, lung, kidney, liver and prostate [4]. Additionally, there is emerging evidence of causing skin lesions, neurotoxicity, cardiovascular diseases, diabetes and negative impacts on foetal and child development [7]. Human exposure to inorganic arsenic occurs mainly by the consumption of groundwater naturally containing high levels of inorganic arsenic, food prepared with this water, food crops irrigated with water sources with high arsenic content or that were treated with phosphate-based fertilizers and pesticides [6,8].

In areas where arsenic is naturally present in high levels, the foods that generally contribute mostly to daily intake are cereals and beans, namely rice [8]. Rice (Oryza sativa L.) feeds approximately 50% of the world population [9] and is one of the most cultivated and consumed cereals in the world [10]. According to the Food and Agriculture Organization (FAO), in 2018, around 517.5 million tons of rice were produced worldwide [11]. While Europe is not self-sufficient in rice production and is the third largest importer in the world, Portugal, along with Italy, Spain and Greece, is one of the European countries with the highest production and consumption of rice per capita [11,12,13]. Portugal produces 160,794 Tonnes of rice per year and the average consumption is 15.9 kg per person per year [14]. 

Rice is a widely used source of carbohydrates during weaning due to its availability, pleasant taste, nutritional value and relatively low allergenic potential. In addition, rice and derived products, such as starch, flour and syrup are used in different baby foods [15]. Rice is not consumed as harvested; it undergoes processing including several stages: Drying to reduce the moisture content of the paddy (harvested rice), cleaning of impurities, removing of the husk, milling to remove hulls and brans (for white rice) and separation of cracked rice (Figure S1, Supporting Information) [16].

The cultivation conditions and the plant’s morphology favour the absorption of arsenic and its accumulation in the grain [1]. The concentration varies according to the soil in which the rice is grown and the type of rice [15]. Numerous investigations have shown that rice grains in arsenic endemic areas contain more than 90% inorganic arsenic [17]. Most of the inorganic arsenic in rice is concentrated in the husk and bran, with concentrations 10 to 20 times higher than the rice grain [15]. Therefore, polished rice is expected to contain lower concentrations of arsenic than whole grains, since the outer layer of the rice was removed [18].

Other authors, worldwide, have reported average concentrations up to 350 µg kg^−1^ of inorganic arsenic [19], with Portuguese rice presenting averages up to 300.8 µg kg^−1^ [20]. Therefore, public health actions are needed to reduce human exposure to arsenic, particularly in areas with naturally high levels in groundwater [8].

In 2015, a regulation was established concerning the maximum levels of inorganic arsenic in foodstuffs, adding limits in rice and rice products. The limit for uncooked white rice is 0.20 mg kg^−1^, for stewed rice is 0.25 and 0.10 mg kg^−1^ for rice for the production of infant food and young children (Table 1) [21].

There are several spectroscopic analytical methodologies for determining metals that allow to extend the scale of concentration of elements to levels of ppm, ppb or even ppt, and perform multi-elemental analysis. The analytical methodologies reported for the determination of inorganic arsenic in rice are in decreasing limits of detection order: Atomic absorption spectrometry with flame (FAAS), with graphite chamber (GFAAS) and with hydride generation (HG-AAS), and inductively coupled plasma mass spectrometry (ICP-MS) [20,22,23]. Additionally, other techniques such as pulse differential voltammetry and square wave voltammetry, both used with the anodic stripping mode are also suitable for the determination of arsenic in rice [24].

This work aimed to detect and quantify inorganic arsenic in different types of rice available in Portugal, such as white vs. brown and long vs. short rice, from diverse production regions. Moreover, the risk assessment was performed evaluating the hazard of the exposure of inorganic arsenic for children and adults.

## 2. Materials and Methods

### 2.1. Sampling

A total of 70 samples were collected; 48 were sampled from a rice factory located in Coimbra District (Portugal) during the months of September and October 2019 and 22 samples were acquired from Portuguese supermarkets in January 2020 (Table S1, Supplementary Materials). The factory samples were collected at the factory entrance, still in shell. To obtain the samples in the proper form for the analysis, it was necessary to proceed with their husking (48 samples) and subsequently the blanching of half of them (24 samples) (Figure S1 Supplementary Materials).

Of the 70 samples analysed 41 (59%) were of white rice, and 29 (41%) of brown rice. Discriminating by size, 22 (31%) samples were of long grain (8 agulha, 10 basmati, jasmine and thai and 4 black, vaporized and wild rice) and 48 (69%) were of short grain (47 were carolino and 1 sushi rice). Sorting by the small Portuguese rice production regions: 39 (56%) with origin in Mondego (between Coimbra and Figueira da Foz), 10 (14%) from the Tejo (between Chamusca and Salvaterra de Magos) and Sado (Alcácer do Sal) river and 21 (30%) samples of unknown origin.

### 2.2. Standards, Chemicals and Materials

Solution of arsenic analytical standard (1003 ± 7.0 mg L^−1^), under the form of H_2_AsO_4_ with 99.999% purity, was purchased from CPAChem (Bogomilovo, Bulgaria) while superpure nitric acid (68%) was acquired from Carlo Erba (Milan, Italy). Ultrapure Milli Q water was daily obtained through a Millipore (Molsheim, France) equipment.

### 2.3. Digestion Procedure and Analysis

In a 50 mL Falcon tube, 5.00 g of ground rice samples were added of 50 mL of nitric acid (1%). After vortexing for 10 min and ultrasound extraction for 15 min it was centrifuged for 15 min, at 2880× *g*. The supernatant was then filtered in a vacuum pump and the extracts were analysed by inductively coupled plasma mass spectrometry (ICP-MS).

Detection of inorganic arsenic was performed on the ICP-MS XSERIES-2 equipment, Thermo Fisher Scientific (Waltham, Massachusetts, EUA), at 1370 W (*m*/*z* 75) (Table S2, Supplementary Materials). A correction equation was used in order to eliminate possible interferences of chlorides present in the samples. Before analysis, all the extracts were diluted 5 times with 0.5% HNO_3_. An internal scandium (Sc) standard (*m*/*z* 45) was used at a concentration of 25 µg L^−1^.

### 2.4. Risk Assessment Calculation

The risk assessment was performed by calculating the percentage of the arsenic intake regarding the selected benchmark dose (BMDL) [25].

The estimated daily intake (EDI) was calculated through a deterministic method [26] using the equation: EDI = (Ʃc) (CN^−1^ D^−1^ K^−1^), where Ʃc is the sum of the compound in the analysed samples (µg kg^−1^), C is the mean annual intake estimated per person of rice and rice products, N is the total number of analysed samples, D is the number of days in a year, and K is the body weight (kg).

According to the last report of the “National Food Survey and Physical Activity, IAN-AF 2015–2016”, the adult population annual on average consumed 25.1 kg of rice per capita and children 19.1 kg year^−1^. As for the 95th percentile, the annual consumption was 47.9 and 62.8 kg year^−1^ for children and adults, respectively [27].

The mean body weight considered for the Portuguese children (2–12 years) and the adult population was 24 and 69 kg, respectively [28].

As for arsenic risk assessment, the EFSA Panel on Contaminants in the Food Chain established a BMDL_10_ (benchmark dose lower confidence limit 10%, representing the lower bound of a 95% confidence interval on a BMD (benchmark dose) corresponding to a 10% tumour incidence) between 0.3 and 8 µg kg^−1^ bw/day for an increased risk of cancer of the lung, skin and bladder, and skin lesions [7]. The risk assessment was performed both for children and adults by comparing the EDI (assuming different scenarios: Average consumption, higher consumption, average contamination, the highest contamination (worst-case scenario)) with the BMDL_10_ value of 0.3 µg kg^−1^ bw/day.

### 2.5. Statistical Analysis

Complete statistical analysis was performed using GraphPad Prism (6.01, GraphPad Software, Inc., San Diego, CA, USA). To test whether the datasets were of Gaussian distribution, D’Agostino–Pearson normality test was used. Since all of the datasets were not normally distributed, with non-homogeneous variances, nonparametric tests were applied. For the evaluation of each type of premade baby food, Kruskal–Wallis test with Dunn’s post-test were used. For the comparison between white and brown rice and for short and long rice samples, Mann–Whitney test was performed. The statistical significance level was set to *p* < 0.05 [29].

## 3. Results

### 3.1. Validation of the Analytical Methodology

Validation was performed to assure the fitness for purpose of the analytical method for the determination of inorganic arsenic in rice (Table S3, Supplementary Materials). Analytical quality control encompassed different performance criteria such as linear range, method detection limit (MDL), method quantification limit (MQL), accuracy and precision features [25]. 

Linearity was studied using standard solutions of arsenic in 0.5% HNO_3_ at the following concentrations: 0.2, 0.4, 0.8, 1.0, 2.0 and 5.0 µg L^−1^, which correspond in the samples to a range of concentrations between 10 and 250 µg kg^−1^. The correlation coefficient obtained was 0.99997. In order to validate the calibration curve, a blank and two standards at 0.2 and 1.0 µg L^−1^ were evaluated, obtaining the results of 0.21 and 1.04 µg L^−1^, respectively.

The MDL and MQL were calculated using the mean and standard deviation of 10 blanks using the formulas: MDL = mean + 3.3 x standard deviation; MQL= mean + 10x standard deviation. The obtained MDL and MQL were 3.3 µg kg^−1^ and 10 µg kg^−1^, respectively.

Five recovery tests were carried out in spiked samples in order to assess the accuracy and precision of the method, with recoveries of 99%, 114%, 106%, 111% and 89% (average of 103.8%). The precision of the method was 10.8%.

For additional quality control, two concentrations of the standard calibration curve (0.2 and 0.8 µg L^−1^) were analysed each 10 samples and at the end of the session. A correction equation was used in the samples to account for the interference of chloride.

Each sample was analysed three times and, to match the analytical methodology criteria, the relative standard deviation in each sample was below 10%, in concentrations over the MQL, and 15% in sample concentrations between the MDL and the MQL.

### 3.2. Occurrence of Inorganic Arsenic in Rice Samples

The total frequency of detection for inorganic arsenic in rice was 81% (57 samples) with an average of 29.3 µg kg^−1^. No sample presented concentrations above the established maximum residue limits (MRLs). However, there was one sample with a concentration that equalled the MRL (100 µg kg^−1^) for rice destined to the production of food for infants and young children.

**Figure 1:**
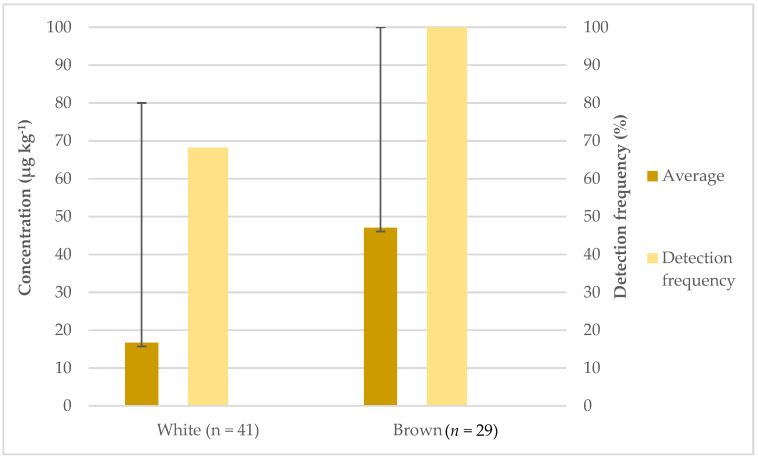
Detection frequency (%), average and maximum concentrations (µg kg^−1^) of inorganic arsenic in white and brown rice.

When comparing white and brown rice, the detection frequency was 100% for brown and 68.29% for white rice (Figure 1). The average concentration of inorganic arsenic in brown and white rice samples was 47.07 and 16.73 µg kg^−1^, respectively. The highest concentration of arsenic was found in one brown rice sample (100 µg kg^−1^). A statistical difference between these two groups was observed, with *p* < 0.0001.

The higher presence of inorganic arsenic in brown rice confirms the data presented by other authors and that the outer layers of rice contain higher concentrations of arsenic [30]. 

**Figure 2:**
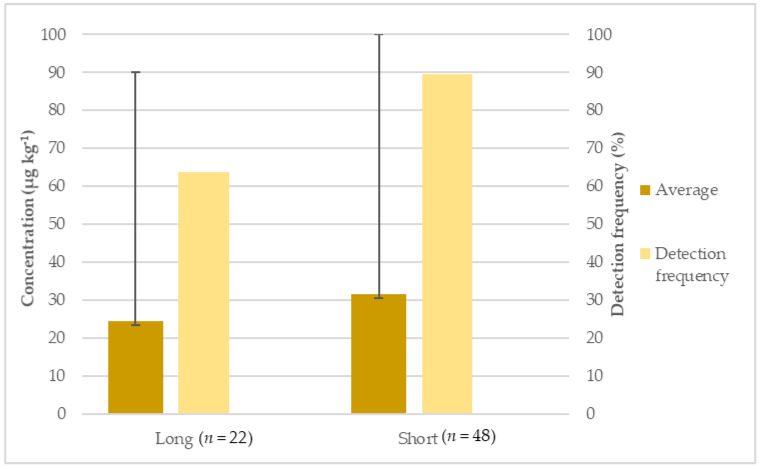
Detection frequency (%), average and maximum concentration (µg kg^−1^) of inorganic arsenic in long and short rice.

Comparing long and short rice, a higher detection frequency (90%) and average concentration (89.6 µg kg^−1^) was found for short rice (Figure 2). On the other hand, long rice presented a detection frequency of 64% and an average concentration of 24.5 µg kg^−1^. A statistical difference (*p* = 0.041) was also found between these two types of rice. While no justification for these results was found, besides the small differences in composition, namely in starch concentration, other authors reported the same pattern [19,31].

**Figure 3:**
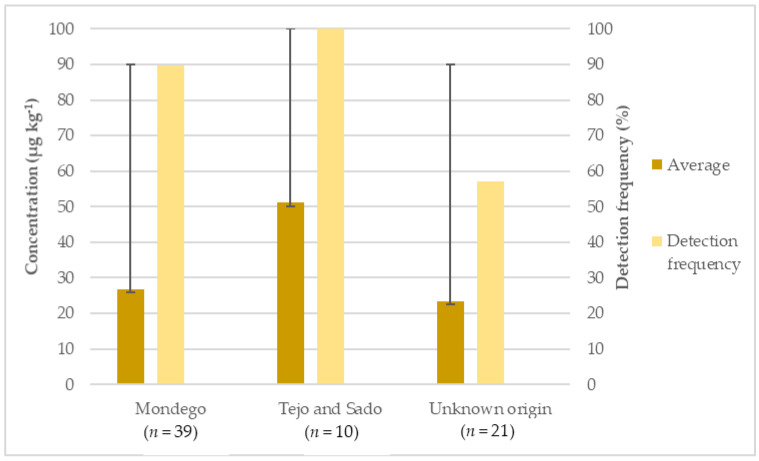
Detection frequency (%), average and maximum concentration (µg kg^−1^) of arsenic in inorganic rice from different origins.

Regarding the origin of the rice, the detection frequency was 100% for the Tejo and Sado, 89.74% for the Mondego and 57.14% for the unknown origin (Figure 3). The group that obtained the highest average concentration originated in the Tejo and Sado followed by Mondego and the samples with unknown origin. There was a statistical difference between the Tejo and Sado group and the unknown origin group, with *p* < 0.0044.

The asymmetries in the obtained results could be related to different soil compositions in terms of rocks and minerals in the different regions and to the climatic conditions that influence the existence to a greater or lesser extent of arsenic in the waters. Additionally, anthropogenic contamination, namely from the use of pesticides and industries along the rivers, can also contribute to arsenic concentration in the near river waters [32].

### 3.3. Comparison with Other Studies

The results obtained in the present study were clearly lower when compared with other scientific published works (Table 2), even when comparing with data from similar regions [20]. From the results presented by other authors we highlight the concentrations found in rice for infant consumption, where levels ranged from 70 to 162 µg kg ^−1^ [23,33,34]. Table 2 shows that, in most of the studied countries, the arsenic concentration in rice frequently surpassed the MRLs. Additionally, in some of them, even the average values were higher than the respective MRLs, namely in Spain and Portugal. This highlights the importance of these studies, since some countries are not complying with the established legislation.

The concentrations obtained for the white rice samples were lower when comparing with other authors where average concentrations varied between 70 µg kg^−1^ and 172 µg kg^−1^ [23,32,35,36]. The same pattern was observed for the brown rice samples, since other authors found concentrations of arsenic between 90 µg kg^−1^ and 230 µg kg^−1^ [19,30]. Additionally, like in the present study, due the arsenic concentration in bran and husk, brown rice presented higher arsenic concentrations.

Comparing the results of short and long rice, the pattern observed by other authors is similar to that found in the present study, with short rice presenting higher average (151 µg kg ^−1^, average of all short rice studies) than long rice (99 µg kg ^−1^, average of all long rice studies) [19,20,22,35].

The countries with the reported highest average concentrations were Spain, with a concentration of 367 ± 4 µg kg^−1^ (medium rice) and 350 µg kg^−1^ (long rice), followed by Portugal with 300.8 µg kg^−1^ (short rice) [19,20]. In contrast, lower average concentrations were reported by India (Punjab), with a concentration of 12.0 µg kg^−1^, and Scotland, with 52 µg kg^−1^ [20,22]. In a previous study carried out in Portugal, inorganic arsenic was determined in rice, with origin in the same regions of Tejo and Mondego. The average concentrations of inorganic arsenic were similar in both regions with samples from Tejo river presenting an average concentration of 224.3 µg kg^−1^ and Mondego with 242.6 µg kg^−1^ [20]. These values are much higher than the ones obtained in the present study, 51.1 µg kg^−1^ for the Tejo River and 26.84 µg kg^−1^ for the Mondego River. These differences are probably due to the different sampling years, separated by five years, which can greatly impact the arsenic concentration in rice.

### 3.4. Risk Assessment

**Figure 4:**
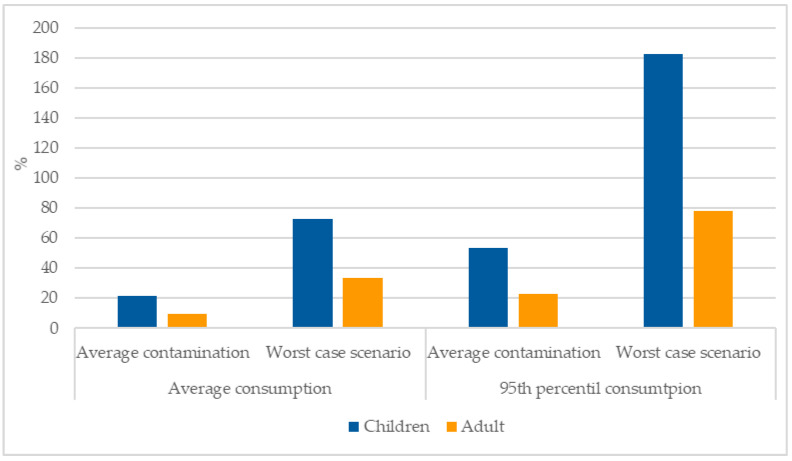
Percentage of the EDI (µg kg^−1^ bw/day) versus the BMDL_10_ (0.3 µg kg^−1^ bw/day).

The risk assessment was performed assuming arsenic concentrations in rice of 29.3 and 100 µg kg^−1^ (average and worst-case scenario, respectively); 19.1 and 25.1 kg (children and adults, respectively) as average annual rice consumption, 47.9 and 59 kg (children and adults, respectively) as 95th percentile of annual rice consumption; and an average weight of 24 and 69 kg (for children and adults, respectively). Using this data, it can be observed that the EDI surpassed the BMDL_10_ of 0.3 µg kg^−1^ of bw/day for children when considering the worst-case scenario contamination and the 95th percentile rice consumption (182.3%) (Figure 4).

Figure 4 shows that when using the worst-case scenario for the concentrations of arsenic found in rice the EDI approaches or surpasses the BMDL_10_, namely for children that have a higher consumption rate of rice per kg of body weight. When using average contamination concentrations, the higher value was 53.4% for children using the 95th percentile of rice consumption.

Using the average exposure to inorganic arsenic for children we can observe that, as expected, the percentage was higher for brown rice (34.2%) than for white rice (12.2%). Moreover, short grain had a higher percentage (22.9%) than short grain (17.8%). Regarding the regions, Tejo and Sado presented clearly higher percentages (37.1%) than Mondego (19.5%) and unknown origin (17.1%).

Observing the present results, there is low risk to consumers through the exclusive consumption of rice, considering that only using the scenario of 95th percentile of rice consumption and the highest contamination found, the children EDI surpassed the lowest BMDL. However, there are groups for which their exposure is higher, thus increasing the risk associated. This is the case for gluten intolerant people, celiacs, for whom the impossibility of consuming cereal-based foods entails rice as an alternative. Rice being the alternative to cereal-based pasta, its consumption is higher by celiacs than that by a non-celiac person. If it is assumed that the consumption of rice by celiacs is approximately double that of a non-celiac person the EDI will also duplicate. 

Additionally, there are also other sources of inorganic arsenic in food (fish, molluscs, water) that should also be included for a more accurate risk assessment [4,7]. This can be observed in certain ethnic groups that have a daily exposure of inorganic arsenic in a diet of about 1 µg kg^−1^ bw/day, and also in high consumers of algae-based products that can have a dietary exposure to inorganic arsenic of about 4 µg kg^−1^ bw/day [7]. These products, namely seaweeds, are becoming part of the Western populations’ diet (consumption of sushi for example), particularly due to their health benefits and essential elements [37,38]. The increased seaweed consumption in the last few years highlights that the exposure to contaminants, namely metals and metalloids, through non-traditional foods is a growing reality that should be accounted for [39].

In 2009, EFSA estimated that national exposures to inorganic arsenic through food and water in 19 European countries ranged from 0.13 to 0.56 µg kg^−1^ bw/day for average consumers, and from 0.37 to 1.22 µg kg^−1^ bw/day for the 95% percentile, values that are higher than the BMDL_10_ (0.3 µg kg^−1^ bw/day) [7]. In 2014, EFSA estimated the average food exposure to inorganic arsenic among infants and the values ranged from 0.20 to 1.37 µg kg^−1^ bw/day. The food exposure average among the adult population (including adults, the elderly) ranged from 0.09 to 0.38 µg kg^−1^ bw/day, values that also surpassed the BMDL_10_. EFSA also confirmed that grains and cereals were the class of food that contributed the most for these values [40].

These values suggest that there are multiple sources of inorganic arsenic in food and water and that the BMDL_10_ value is easily surpassed by the EDI on several occasions. This highlights the importance of determining the inorganic arsenic in food and water, namely on grains and cereals, the main contributors for EDI of this contaminant.

## 4. Conclusions

Organic and inorganic arsenic is mainly present in water, originating from different sources, with foods that are irrigated with large amounts of water, such as rice, accounting for a greater exposure when compared to other cereals.

In this study, the identification and quantification of inorganic arsenic in rice was performed by ICP-MS. The methodology used proved to be adequate, allowing for an MDL of 3.3 µg kg^−1^ and a MQL of 10 µg kg^−1^, while the accuracy of the method ranged between 89% and 114%.

The results obtained showed that of all the analysed samples contained inorganic arsenic, however, none above what is stipulated by law for inorganic arsenic present in rice. It is also concluded that the brown rice samples are the ones that present the highest average of inorganic arsenic (47.1 µg kg^−1^). Short rice had higher average concentration (31.5 µg kg^−1^) than long rice (24.5 µg kg^−1^). Among the different regions of origin in Portugal Tejo and Sado region presented the highest amount of inorganic arsenic (51.1 µg kg^−1^). 

Considering the risk assessment carried out, it can be concluded that only in very specific cases (children in the 95th percentile of rice consumption and worst-case scenario concentration) the BMDL_10_ (0.3 µg kg^−1^ of bw/day) is surpassed by the EDI. 

It should be noted that rice is not the only source of inorganic arsenic. Therefore, other sources that also contribute to the daily intake should also be considered for a correct risk assessment. Additionally, there are population groups that present a higher risk to the exposure of inorganic arsenic like children, celiac people, some ethnic groups and high consumers of algae-based products that can highly exceed the BMDL_10_.

## Figures and Tables

**Table 1:** Maximum residue limits (MRLs) for arsenic (inorganic) in rice and rice products (Regulation 2015/1006).

Foodstuff	Maximum Residue Limit (mg kg^−1^)
Non-parboiled milled rice (polished or white rice)	0.20
Parboiled rice and husked rice	0.25
Rice waffles, rice wafers, rice crackers and rice cakes	0.30
Rice destined for the production of food for infants and young children	0.10

**Table 2:** Occurrence of arsenic in rice across the world.

Type of Rice	Country	Year	Number of Samples	Detection Frequency (%)	Range and Standard Deviation (µg kg^−1^)	Mean Concentration and Standard Deviation (µg kg^−1^)	References
Long	Scotland ^a^	2014	44 ^b^	100	NA	94 ± 1	[22]
	Scotland ^a^	2014	44 ^b^	100	NA	52 ± 10	[22]
	Scotland ^a^	2014	44 ^b^	100	NA	61 ± 4	[22]
Long-Agulha	Spain	2007	39 ^b^	97	NA	350 ± 16	[19]
	United States of America	2003	40 ^b^	NA	33–271	NA	[33]
Long-Agulha brown	Spain	2007	39 ^b^	97	NA	230 ± 20	[19]
Long-Basmati	Scotland ^a^	2014	44 ^b^	100	NA	53 ± 7	[22]
	Scotland ^a^	2014	44 ^b^	100	NA	69 ± 9	[22]
	Punjab, India	2014	10 ^b^	100	NA	12.0 ± 5.48	[20]
	India	2007	39 ^b^	97	NA	67 ± 1	[19]
	Belgium ^a^	2018	5	100	19 ± 8–48 ± 21	30 ± 10	[35]
Long-Basmati Brown	Spain	2007	39 ^b^	97	NA	148 ± 4	[19]
Long-Steamed	Iberian Peninsula	2016	11	100	22–70	83 (median)	[30]
Long-Thai	Thailand	2007	39 ^b^	97	NA	175 ± 8	[19]
	Belgium ^a^	2018	7	100	63 ± 16–147 ± 37	77 ± 32	[35]
Long-Thai jasmine	Scotland ^a^	2014	44 ^b^	100	NA	64 ± 3	[22]
	Scotland ^a^	2014	44 ^b^	100	NA	62 ± 3	[22]
	NA	2014	10 ^b^	100	NA	85.5 ± 10.39	[20]
	Thailand	2014	10 ^b^	100	NA	62.6 ± 6.21	[20]
Medium grain	Spain	2007	39 ^b^	97	NA	367 ± 4	[19]
	United States of America	2003	40 ^b^	NA	46–114	NA	[33]
Medium grain-Brown	Spain	2007	39 ^b^	97	NA	145 ± 5	[19]
NA-Baby rice	United Kingdom	2014	29	100	63–268	NA	[30]
NA-White	Switzerland ^a^	2018	27	NA	5.6–188	94	[36]
	New Zealand and Australia ^a^	2019	36	100	40–100	70	[23]
	Belgium ^a^	2018	7	100	67 ± 18–245 ± 64	172 ± 81	[35]
NA-Brown	Scotland ^a^	2014	44 ^b^	100	NA	137 ± 5	[22]
	Iberian Peninsula	2016	20	100	53–47	157 (median)	[30]
	Switzerland ^a^	2018	4	NA	117–172	152	[36]
	New Zealand and Australia ^a^	2019	21	85	<20–120	90	[23]
	Belgium ^a^	2018	5	100	119 ± 32–243 ± 67	167 ± 47	[35]
NA-Infant Rice	China	2011	14	100	52–247	114 ± 15	[34]
	USA	2011	5	100	93–159	125 ± 14	[34]
	United Kingdom	2011	5	100	107–267	162 ± 29	[34]
	Spain	2011	7	100	10–111	85 ± 10	[34]
	New Zealand and Australia ^a^	2019	15	100	40–140	70	[23]
NA-Polished	Iberian Peninsula	2016	113	100	27–75	71 (median)	[30]
Short-Carolino	Tejo, Portugal	2014	10 ^b^	100	NA	300.8 ± 31.79	[20]
	Mondego, Portugal	2014	10 ^b^	100	NA	242.6 ± 32.97	[20]
	Portugal	2014	10 ^b^	100	NA	217.5 ± 15.94	[20]
	Tejo, Portugal	2014	10 ^b^	100	NA	224.3 ± 32.97	[20]
Short-Japanese	Scotland ^a^	2014	44 ^b^	100	NA	99 ± 5	[22]
Short-Paella	Scotland ^a^	2014	44 ^b^	100	NA	70 ± 3	[22]
	Scotland ^a^	2014	44 ^b^	100	NA	67 ± 3	[22]
Short-Risotto	Scotland ^a^	2014	44 ^b^	100	NA	120 ± 18	[22]
	Scotland ^a^	2014	44 ^b^	100	NA	114 ± 10	[22]
	Italy	2014	10 ^b^	100	NA	53.2 ± 8.21	[20]
Long	Scotland ^a^	2014	44 ^b^	100	NA	94 ± 1	[22]
	Scotland ^a^	2014	44 ^b^	100	NA	52 ± 10	[22]
	Scotland ^a^	2014	44 ^b^	100	NA	61 ± 4	[22]
Long-Agulha	Spain	2007	39 ^b^	97	NA	350 ± 16	[19]
	United States of America	2003	40 ^b^	NA	33–271	NA	[33]
Long-Agulha brown	Spain	2007	39 ^b^	97	NA	230 ± 20	[19]
Long-Basmati	Scotland ^a^	2014	44 ^b^	100	NA	53 ± 7	[22]
	Scotland ^a^	2014	44 ^b^	100	NA	69 ± 9	[22]
	Punjab, India	2014	10 ^b^	100	NA	12.0 ± 5.48	[20]
	India	2007	39 ^b^	97	NA	67 ± 1	[19]
	Belgium ^a^	2018	5	100	19 ± 8–48 ± 21	30 ± 10	[35]
Long-Basmati Brown	Spain	2007	39 b	97	NA	148 ± 4	[19]
Long-Steamed	Iberian Peninsula	2016	11	100	22–70	83 (median)	[30]

NA-Not available; ^a^—Samples purchased at supermarkets in the respective countries, without information on the country of origin of the sample; ^b^—Total of the studied samples, not specific to the type of rice.

## Data Availability

Not applicable.

## Supplementary Materials

The following supporting information can be downloaded at: https://www.mdpi.com/article/10.3390/foods11030277/s1, Table S1: Sample characterization; Table S2: Arsenic detection settings, in the ICP-MS XSERIES-2, ThermoUnican equipment; Table S3: Validation results for the analytical methodology; Figure S1: Preparation of white (A) and brown (B) rice samples.
