# Supplementary material for: Arsenic in Portuguese Rice: Is There Any Risk?
Source: Foods. 2022 Jan 20;11(3):277. doi: 10.3390/foods11030277 (PMC8834159; doi:10.3390/foods11030277)
Supplement: Supplementary file 1 [file foods-11-00277-s001.zip › foods-1544511-supplementary.pdf]

Supporting information for:
Arsenic in Portuguese rice. Is there any risk?

Alexandra A. Silva [a], André M.P.T. Pereira [a,*], Liliana J.G. Silva [a], Angelina Pena [a]

[a] *LAQV, REQUIMTE, Laboratory of Bromatology and Pharmacognosy, Faculty of Pharmacy, University of Coimbra, Polo III, Azinhaga de St[a] Comba, 3000-548 Coimbra, Portugal*

***Corresponding author:**
LAQV, REQUIMTE, Group of Bromatology, Pharmacognosy and Analytical Sciences, Faculty of Pharmacy, University of Coimbra
Pólo das Ciências da Saúde, Azinhaga de Santa Comba, 3000-548 Coimbra, Portugal.
Tel: +351239488400
Fax : +351239827126

E-mail addresses:
alexandra.silv@hotmail.com (A.A. Silva), amptpereira@gmail.com (A.M.P.T. Pereira), ljgsilva@hotmail.com (L.J.G. Silva), apena@ci.uc.pt (A. Pena)

Figure S1. Preparation of white (A) and brown (B) rice samples.

A)

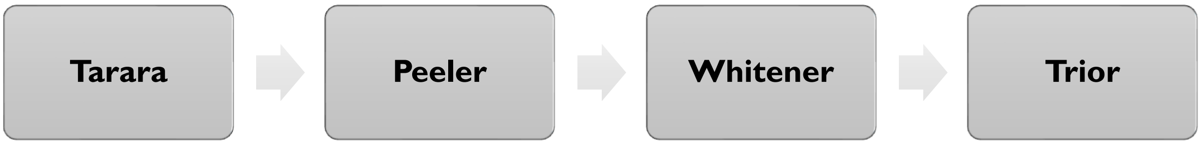

B)

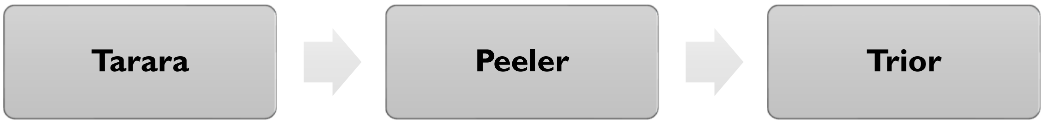

The tarara separates the rice from residues that it can bring, such as straw; peeler removes the husk from the rice; in the whitener the rice under goes a physical process of friction resulting in the removal of the aleurone layer; in the trior the whole rice is separated from the cracked one.

Table S1. Sample characterization.

Type of rice	Geographic origin	White / Brown rice	Brand
**Short - Carolino**	Mondego	White	NA
**Short - Carolino**	Mondego	Bronw	NA
**Short - Carolino**	Mondego	White	NA
**Short - Carolino**	Mondego	Bronw	NA
**Short - Carolino**	Mondego	White	NA
**Short - Carolino**	Mondego	Bronw	NA
**Short - Carolino**	Mondego	White	NA
**Short - Carolino**	Mondego	Bronw	NA
**Short - Carolino**	Mondego	White	NA
**Short - Carolino**	Mondego	Bronw	NA
**Short - Carolino**	Mondego	White	NA
**Short - Carolino**	Mondego	Bronw	NA
**Short - Carolino**	Mondego	White	NA
**Short - Carolino**	Mondego	Bronw	NA
**Short - Carolino**	Mondego	White	NA
**Short - Carolino**	Mondego	Bronw	NA
**Short - Carolino**	Mondego	White	NA
**Short - Carolino**	Mondego	Bronw	NA
**Short - Carolino**	Mondego	White	NA
**Short - Carolino**	Mondego	Bronw	NA
**Short - Carolino**	Mondego	White	NA
**Short - Carolino**	Mondego	Bronw	NA
**Short - Carolino**	Mondego	White	NA
**Short - Carolino**	Mondego	Bronw	NA
**Short - Carolino**	Mondego	White	NA
**Short - Carolino**	Mondego	Bronw	NA
**Short - Carolino**	Mondego	White	NA
**Short - Carolino**	Mondego	Bronw	NA
**Short - Carolino**	Mondego	White	NA
**Short - Carolino**	Mondego	Bronw	NA
**Short - Carolino**	Mondego	White	NA
**Short - Carolino**	Mondego	Bronw	NA
**Short - Carolino**	Mondego	White	NA
**Short - Carolino**	Mondego	Bronw	NA
**Short - Carolino**	Mondego	White	NA

**Short - Carolino**	Tejo	White	NA
**Short - Carolino**	Tejo	Bronw	NA
**Short - Carolino**	Tejo	White	NA
**Short - Carolino**	Tejo	Bronw	NA
**Short - Carolino**	Tejo	White	NA
**Short - Carolino**	Tejo	Bronw	NA
**Short - Carolino**	Tejo	White	NA
**Short - Carolino**	Tejo	Bronw	NA
**Short - Carolino**	Sado	White	NA
**Short - Carolino**	Sado	Bronw	NA
**Short - Carolino**	NA	White	Ceifeira
**Short - Carolino**	NA	White	Golden Sun
**Short - Sushi**	NA	White	Caçarola
**Long - Agulha**	Mondego	White	NA
**Long - Agulha**	Mondego	Bronw	NA
**Long - Agulha**	Mondego	White	NA
**Long - Agulha**	Mondego	Bronw	NA
**Long - Agulha**	NA	White	Caçarola
**Long - Agulha**	NA	White	Amanhecer
**Long - Agulha**	NA	White	Por si
**Long - Agulha**	NA	White	Pingo Doce
**Long - Basmati**	NA	White	Por si
**Long - Basmati**	NA	White	Caçarola
**Long - Basmati**	NA	White	Pingo Doce
**Long - Basmati**	NA	White	Oriente
**Long - Basmati Brown rice**	NA	Bronw	Continente Seleção
**Long - Jasmine**	NA	White	Auchan
**Long - Jasmine**	NA	White	Pingo Doce
**Long - Thai**	NA	White	Caçarola
**Long - Thai Jasmine**	NA	White	Continente
**Long - Punjab Aromatic**	Punjab	White	Continente Seleção
**Long - Steamed**	NA	Brown	Por si
**Long - Black**	NA	Brown	Caçarola
**Long - Wild**	NA	Bronw	Caçarola
**Long - Brown rice**	NA	Bronw	Saludães

NA – Not available

Table S2. Arsenic detection settings, in the ICP-MS XSERIES-2, ThermoUnican equipment.

**Survey Setup**	Sweeps	5
	Dwell Time	600
	Channels per mass	10
	Acquisition Duration	6623
**Main Run Setup**	Main Run	Peak Jumping
	Sweeps	200
	Dwell Time	10000
	Channels per mass	1
	Acquisition Duration	22287
	Channel Spacing	0,02
**Settings sets**	Extraction	-106,00
	Lens 1	-1170,00
	Lens 2	-79,20
	Lens 3	-185,90
	Pole Bias	-3,00
	Sampling Depth	108,00
	Nebuliser	0,87
	Forward power	1370,00

Table S3. Validation results for the analytical methodology.

	**Standard linearity (0.2 – 5.0 µg L^{-1}) (r^2)**	**MDL**	**MQL**	**Recovery assays 10.0 µg kg^{-1} (range) (n=5)**	**Precision**
**Arsenic result**	0.99997	3.3 µg kg^{-1}	10 µg kg^{-1}	89-114%	10.8%

MDL – Method detection limit; MQL – Method quantification limit
